# Report on Cerebro-Spinal Meningitis

**Published:** 1866-11

**Authors:** J. S. Jewell

**Affiliations:** Prof. Anatomy, Chicago Medical College; Member of Illinois State Medical Society, etc.


### CHICAGO MEDICAL EXAMINER.

N. S. DAVIS, M.D., EDITOR.

VOL. VII. NOVEMBER, 1866. NO. 11.

### Original Contributions.

ARTICLE XXXVIII.

### REPORT ON CEREBRO-SPINAL MENINGITIS.

BY J. S. JEWELL, M.D., Prof. Anatomy, Chicago Medical College, etc.

[CONCLUDED.]

From this summary, which is made up from the facts collected from various authentic sources, it will be perceived that the diseases just considered are *not* identical, but they really present wide differences, such as can leave no doubt of their *non*-identity. This parallel requires no discussion to evolve its teachings. While I have no doubt that cerebro-spinal meningitis may have imparted to it peculiar hues, so to speak, or tendencies, by the typhus, malarial, or erysipelatous "constitutions," or may be complicated with pneumonia or other special forms of disease, I feel I am warranted in concluding this to be a disease *sui generis*, and not a mere form of typhus, as it has been assumed to be.

If it has been shown that cerebro-spinal meningitis and typhus are not identical, it will hardly be worth while to show that the opinions above referred to, as to its identity with other forms of disease, have no sufficient foundation in fact.

In the third place, the evidence does not show that it is contagious. So far as the vast *majority* of cases are concerned, they are clearly not contagious in their origin. A large number of cases, however, are detailed by HIRSH, BOUDIN, RUM-

MEL, and others, which *seem* to show the disease is contagious. I will not here give the particular testimony on which its contagiousness is affirmed, but will remark on that evidence, that in diseases like the present, where comparatively only a few cases have occurred which favor the idea of contagion, we may well scrutinize them closely, before we admit them.

In diseases like small-pox, *e.g.*, when, at least in the majority of cases, we can show exposure to contagious influence clearly to have *preceded* the occurrence of the disease, and in which we can clearly communicate the disease by contact or inoculation, and in which it presents a series of distinct stages and is self-limited, I say in *such* cases, there can be no doubt. But it is not so in this disease. If we *admit* contagion, it is clear the majority of cases do not occur from it, but under circumstances forbidding such a conclusion. Indeed, there is, perhaps, not a single law of contagion to which it conforms only in seeming, while it *does* conform to the general facts or laws of epidemic influence.

In determining the action of contagion in such a case as this, it can only be done on the observed order of sequence. To be valid, it should be shown in the majority of cases. But here, on the contrary, comparatively only a few have been observed. In the majority of cases, no such order has been observed or has existed. Besides, in those cases in which it *has* seemed to operate it has not been shown that such cases were not among people who were living under the operation of the same cause or causes, which, producing disease in *one*, really (as seems most probable) produced the same disease in due time in others. The evidence which has been offered in support of the belief in contagion, can only yield that support by a palpable *petitio principii.* On the whole, the evidence seems to me not to support the question of contagion.

Is the disease infectious? Most authors decide, or seem inclined to decide, it is. There has always seemed to me more than the usual looseness with which medical terms are employed, in the use of the terms "contagion" and "infection." I will state what I understand these terms to mean:—

By *contagion*, I understand such a positive cause of disease as, emanating from a sick person, may be communicated *through the air*, to *another* person, so as to produce in such person the same form of disease. As a good example, I would mention small-pox.

By *infection*, I mean a positive cause of disease, such as may be communicated from a person diseased, by *contact*, to another person not diseased, and which is capable when thus communicated, as in the case of contagion, of producing or reproducing the same form of disease which was the occasion of it. As examples, I would mention syphilis, hydrophobia, etc., diseases which are *not known* to be communicated through the air or by contagion, but *are* known to be communicated by contact.

But some diseases are both contagious and infectious, as small-pox, erysipelas, and, *probably*, typhus. I would call those diseases contagious only which are clearly capable of affecting persons through the medium of the air, in the way already mentioned. Those infectious only which are capable of being propagated in the way mentioned above. Such diseases as have their *causes* communicated by contact, and in this way are propagated, as in scabies or porrigo, rather than the *products* of diseased action, as in small-pox, hydrophobia, syphilis, etc., *might* or might *not* be called infectious, according to one's pleasure. If this be a correct definition of infection, I must say there is no evidence worthy of note that the disease is infectious.

Having, in some measure, seen what the disease is *not*, let us now turn to the direct and important question:—What does the evidence teach us the disease *is?* What is its *seat*, its *nature?* These questions will be found difficult as they are important.

Having concluded the essential cause is external, it now becomes us, in view of its nature, to trace its effects as observed in the blood and textures of the body, and, as near as possible, the *order* in which they appear, and, if *possible*, *how* the phenomena are produced. It will be admitted that the cause, whatsoever it is, must first enter the blood. Here, the blood may serve simply the purpose of a vehicle, conveying the poison to the parts on which it acts; or the blood may form the *nidus* for the development of the poison in quantity sufficient to produce its visible effects, as in small-pox or hydrophobia, probably; or the poison (if we may so call it for the time) may initiate a series of changes in the blood which, in their turn, become the cause of the observed phenomena. Some of the facts already given will be called up in the statements which follow, but for a different purpose than before. This statement will, I hope, save me from the charge of thoughtless repetition.

That the poison, whatsoever it may be, *sometimes* acts directly on the nervous system, seems to be matter of fact. It must be distinctly remembered that the question is not whether there *are* changes in the blood, but whether those changes become the cause or causes of the symptoms and appearances presented in this disease.

Now, though we are not able to say positively, *no* blood-changes have occurred sufficient to account for the disease, its sudden attack, or speedy fatal results, yet, on the other hand, our *experience* in regard to blood-changes in other diseased states is opposed to a belief that death is due to changes in the blood, rather than to a poison suddenly introduced into, or developed in the blood, though independent of it. Our *experience* (and this is all we can rely on here) makes it easier for us to believe the latter than the former. There are many obvious instances where known poisons have been introduced into the blood and have caused death in as short a time, in a way not very dissimilar, and which have left behind no greater traces of their action than some of the reported cases have of sudden death in this disease. It is difficult, with our present experience, to imagine blood-changes so grave take place in a time so *short* as the suddenness of the attack requires they often should. I find it difficult, if not impossible, to resist the conviction that some energetic poison *has been* introduced into the blood and has acted directly on the nervous system, sufficiently concentrated or violent, in some cases, to destroy life speedily, by destroying the energy or power of the organic nervous system, striking thus at the very fountain of life; or, without acting in this marked manner, initiating a series of changes in the blood and tissues, which, in process of time, and according to circumstances, may result in death or recovery. I am reminded very much of the action of hydrocyanic acid, strychnia, morphia, etc., when I witness or consider such cases.

As already remarked, we do not *know* there have been no changes in the blood sufficient to account for the phenomena of this disease, but what we *do* know does not render such probable. But whatsoever uncertainty there may be about this, the evidence goes to show the nervous system as earliest and most profoundly affected, both sympathetic and cerebro-spinal, especially the former, and the symptoms are, in great measure, confirmed by the *post mortem* appearances.

The sudden and invariable reduction of temperature, and the change in the character of the pulse, always indicative of a loss of energy or power, and perhaps other symptoms, point at once to the organic nervous system as the part primarily affected, so far as the *earliest symptoms show.* We know enough of the power and influence of this nervous system in the organic processes or actions of the body, and especially those comprehended under the title of nutrition, not to be surprised that any grave affection *it* should experience would be attested, or followed in due season, by such marked phenomena as even *this* disease presents.

After a careful consideration of the phenomena, the conclusion arrived at seems to me most in accordance with them, *viz.:* The organic nervous system (and, probably, at the same time the cerebro-spinal) is acted on in various degrees by some subtle epidemic cause, unknown save in its effects, which directly impairs its energy, and, through it, probably initiates those changes in the blood and textures to which many of the symptoms during life and appearances after death are due.

Whether the blood is primarily affected, other changes following as consequences, is true or not, we have seen that changes of a marked character do occur, and that they are, probably, not due to retained excretory matter, or to a deprivation of some of its appropriate elements, we have already seen. During life, the blood is characterized by unusually bright color, by firm coagulation, by large clot, often by a buffy coat, and by an increase of fibrine and corpuscles; after death, by fluidity and dark color, though not always the former.

As regards the fibrine, in whatsoever way we account for its increase, or whatsover effects we may attribute to it, it seems probable it is owing to its excess we have the remarkable tendency to plastic exudation on the surface of the brain and spinal cord, where time has been afforded for it to occur. One reason *why* this effusion takes place so freely on this surface seems to be, that it is so surpassingly rich in vessels, on the one hand, and that they are *so superficial*, on the other hand. Whether the fibrine plays any other important part than this, seems mainly matter of conjecture.

As regards the red corpuscles, whether it is negative, as by a failure to disintegrate, or positive, as by reason of increased production, and as to what share they may have in producing the phenomena of the disease, in relation to all such points we have no very definite information.

Albumen, as we have seen, has been found in the urine in many cases, especially in the later stages or during convalescence. But the absence of dropsies, except to a limited extent, and of all the symptoms usually connected with the blood impoverished of its albumen, would lead one not to lay much stress on *this* feature. Whether the salts are less in quantity than normal in the blood the facts do not show.

It will be remarked of these blood-changes, so far as they have been matter of observation, they have been witnessed only after several days of the disease have passed.

That in cases which have had some duration, the blood after death is darker and more fluid than it is customary to find it, may be significant. But these same conditions, so far as we can tell, exist, on the average, in a more perfect degree in forms of disease widely different from this, and, besides, as we have seen, they do not obtain in all cases. It must be remembered, the blood during life does not show anything of such a state, but the contrary. It is no uncommon thing, in fact, to find a similar state of the blood in cases where death has occurred from violent diseases.

The view I should be inclined to take of the blood in this disease, is, that whatsoever changes appear in it constitute simply a part, and a very important part, of the disease, and that these changes are probably subsequent, in order of time, to the affection of the organic nervous system. The diseased states of the blood may; and probably *do*, have an important causative relation to some of the later phenomena of the disease.

If we take all the leading phenomena of the disease, from beginning to end, and regard simply the direction they point, it will be readily seen they point almost unanimously to the nervous system. The chilliness, the state of the pulse, the sympathetic vomiting, the mental disturbance, the disturbance of general and special sensibility and of motion, all such symptoms point to the nervous system as the *characteristic seat* of the disease.

Having thus reviewed the symptoms and blood appearances, and having, in some degree, ascertained the truths to which they seem to point, let us now turn to the pathological appearances and interrogate *them*, and find if their teachings correspond to those the symptoms have apparently afforded.

When we consider the *post mortem* appearances, we find, excepting those found within the cerebro-spinal cavity, none sufficiently constant to be considered as properly belonging to the disease. They are simply incidental, or throw no light on the disease not already given in those observed in the cerebro-spinal cavity. This being admitted, and the blood appearances having already been considered, our attention will be confined to the central nervous system and its envelopes.

As regards the contents of this cavity, they have never been found wholly free from at least some evidence of disease. The most constant, the uniform morbid appearance, has been congestion.

As regards exudations, they always seem to be present, where time has been given for them to appear in noticeable quantity. But before we consider the character of the effusion, let us see clearly where the *post mortem* appearances occur.

The *dura mater* is probably always either congested or has abnormal fulness of its sinuses. It is almost never structurally diseased or exhibits in a noticeable degree a plastic effusion.

The *arachnoid* is also very seldom diseased, especially on its outer surface. It is many times somewhat discolored, roughened, and thickened as we should expect a delicate membrane like this to be, bathed on one surface in exuded matter. It is *very seldom* structurally diseased. Those who will remember how sparsely this membrane is supplied with vessels and nerves will not be much surprised at this. The contents of its cavity seldom present marked evidences of disease.

But when we turn to the pia mater, the case is changed. It is *never* free from some evidence of disease, and in almost all cases evidence the most indubitable, such as marked effusion, plastic and otherwise; if not *this*, congestion. The effusion, in such places and to such extent as has been already indicated in the *post mortem* history. Now, this effusion is almost uniformly spoken of as occurring on the serous surfaces of the body, for which the disease is believed to have a decided preference, especially the serous surface within the cerebro-spinal cavity. But it so happens, as regards the latter, that it almost never is the seat of the disease. The *only* part constantly diseased is the pia mater, which is not a *serous* membrane any more than the periosteum is.

It will be remembered that this membrane is simply a fibrous investment for the brain and spinal cord, being thickest on the latter. It forms not only an investment for the brain and cord, but a trellis or framework, so to speak, in the meshes of which the arteries destines to enter the cerebral substance ramify and subdivide to the requisite degree of fineness. It is *fibro-vascular*, and *not* serous. Its extreme richness in bloodvessels, and their *superficial character*, will probably, in some measure, explain why this membrane is so often the seat of disease, and why the effusion occurs on it in such abundance. Something may be due to the fact, that the cerebral circulation occurs in an unyielding airtight case, in this respect being different from the circulation elsewhere, save in the bones, perhaps. I have long thought the conclusions reached by Dr. Burrows, regarding the cerebral circulation, were not altogether sound, and that the fact should be held as important that the cerebral circulation takes place under the conditions it does. In this connexion, I merely suggest this as a matter to be thought of in explaining, or seeking to explain, the phenomena of the disease now under consideration.

Another fact is worthy of note, that the effusion is most abundant on those parts most vascular, as on the under surface of the brain, where the pia mater covers the under surface of the great ganglia which are richly supplied with vessels.

But why the *cause* exerts its influence *here* so constantly, can only be explained when we are able to explain why opium acts on the brain, strychnia on the spinal cord, one medicine on this part, and another on that. Whatsoever peculiar effects such medicines produce, they stand to such effects in the relation of cause, it is almost superfluous to say. In this disease we have certain *effects;* whatsoever the essential cause may be, they are, in the main, *its* peculiar effects. It is no more difficult for us see how the supposed cause of this disease shall produce its characteristic effects, than for us to explain to ourselves why the medicines named should act as they do, rather than some other way or place.

It now becomes a question, whether this supposed cause acts on the brain and spinal cord directly or indirectly. I am prepared to admit its direct action on the brain and spinal cord. But whether this be true or not, I deem it possible to show, as highly probable, that the results which appear within the cerebro-spinal cavity are due, in no small degree, to the state of the organic nervous system. I will shortly return to this topic.

In cases where effusion has occurred to any considerable extent it is not difficult to account for the coma and many of the other symptoms, more especially when the brain and spinal cord are substantially diseased, as they often are. The derangements of the nutrition and of structure, the pressure, and the like would be considered sufficient causes for death without the direct effect of the supposed poison.

As regards the character of the effusion, generally speaking, it resembles what we find in other congestions and inflammations so closely, that no difference except in degree is to be perceived. The exuded matter has in many epidemics appeared purulent in a marked degree, as, indeed, we would occasionally expect it to be in any epidemic. Though this purulent character is more common in this disease than other forms of inflammation of the same parts, yet it is by no means *peculiar* to this disease. The most that can be said is, that the disease shows a more decided tendency to the exudation or formation of pus than ordinary. This will be an interesting point to bear in mind in the therapeutical management of the disease.

There are several points which have been established, with various degrees of certainty, by recent anatomical and physiological investigations, which, if held in view, will assist us in understanding or interpreting the phenomena of diseases affecting the nervous system. Among them may be mentioned, that it is pretty well established that the cerebrum or the gray matter on its surface is the seat of those automatic actions such as we become *conscious* of *willing*, called *volitions*, and also that part of the brain immediately concerned in intellection, and to be the part wherein we become conscious of sensory impressions. All sensations, as such, are recognized here; all volitions, as such, originate here. Beneath this great primary centre, and subordinate to, and partly imbedded in it, are certain other centres, such as the corpora striata, which are probably the centres to which all *voluntary* impulses converge from the surface of the hemispheres, previous to their distribution to the voluntary muscular system; the thalami optici, to which converge, probably, all *general sensory* impressions, previous to being transmitted to the cerebral hemispheres for recognition; and the cerebellum, which has, probably, as its chief function, the coördination of the voluntary muscular movements. About these *latter*, are massed the various centres for the special senses. I say, by bearing such facts in mind, we will not be surprised to find derangements, in various degrees, of the functions those parts seem destined to perform, when we find, as we do in *post mortem* examinations, they are so frequently diseased, or find evidence of disease in their immediate vicinity. By remembering such facts, we are many times able to connect *post mortem* appearances with the symptoms of the disease, or, conversely, to determine, with more or less certainty, from the symptoms, the situation and extent of the pathological changes going on during life, a very desirable thing to accomplish when possible.

But, returning from this digression, let us now recur to the question, as to what share the state of the organic nervous system has in producing the diseased or abnormal condition of the circulation, and the consequences which flow from it, as regards the cerebro-spinal axis.

This cannot well be shown, without a reference to certain facts and experiments, and to certain views which I think the facts warrant, which, though it may be a digression, they are, however, of sufficient importance to justify their introduction in this place. Neither the facts nor the views can be said to be new, but their importance has never been sufficiently recognized, so far as I know.

* At one end of the elastic arterial tube is a powerful muscular appartus, the heart, by which this elastic tube is kept constantly distended, and which communicates, in that class of animals possessed of it, the original impulse to the blood. Part of the force exerted by the heart is expended in setting the blood in motion; part, in opposing the elasticity of the arteries, by which latter the force so expended is *conserved*, and in the interval between one ventricular contraction and the next which succeeds it, is expended mainly in reacting on the contained column of blood. In obedience to this force, the blood being prevented from returning toward the heart, is forced onward steadily toward the distal end of the artery. By the time the blood has reached the distal end of the artery, its momentum has become so much diminished by friction and the like that it could probably not be circulated through the numberless small, winding capillary passages, were it not for some

* These same views are more fully presented in a paper to be found in the August No. of the *Chicago Medical Examiner.*

arrangement, either at the distal end of the arteries or in the capillaries, for communicating a new impulse to the blood which has just entered, or is about to enter, the capillaries. But the capillaries have been shown not to be muscular. They have very thin and delicate walls, just sufficient, apparently, to separate the blood within from the tissue without the vessel. If *anywhere*, the muscular structure for communicating this new impulse to the blood must be nearer the heart. Now, it has been demonstrated that the small arteries or arterioles have muscular walls. Manifestly, this is not the place to refer to all the experiments which have been or may be made to prove this fact; but I will refer to one or two:—

The brothers WEBER found, upon drawing out the mesentery of a frog and placing it under the microscope, if they transmitted through one of the small arteries a galvanic current, it immediately contracted, and by continuing the current, the artery would diminish in size *very much;* but if the current was too *powerful*, or too *long-continued*, the artery ceased to contract, and continued to dilate until much greater than the natural size—the muscular tissue, in the last case, losing its power to contract on the application of appropriate stimuli. The same phenomena may be observed, even more strikingly, in the wing of the bat, with the additional advantage that it is a warm-blooded animal.

We will, then, admit the muscularity of the small arteries, and that they readily contract on the application of stimuli, as other muscular organs do, and that this muscular tissue may be paralyzed from excessive stimulation, or from the want of it.

Another question now arises, whether these small arteries, like other muscular organs, from which they differ essentially only in size, are supplied by nerves. They are, as has been and can be demonstrated, and especially do these small arteries receive twigs from the organic nervous system.

Now, are there any circumstances known, by which we can know the muscular coat of these vessels to act under a stimulus from this nervous system. If we reason from analogy, they do. When we take a swallow of water or a morsel of food into the esophagus, for example, it immediately excites, by reflex action through some of the neighboring ganglia of the sympathetic system, contraction of the esophagus, by which the fluid is propelled along in a certain direction.

The only difference worthy of notice in the two cases seems to be that of size. You have in both cases a muscular tube supplied by twigs from the organic nervous system and a fluid, filling it rythmically, or with an impulse. How can we refuse to admit contraction in the one case, when it is plain it occurs in the *other* case? If this muscular apparatus is not here for some such purpose as this, what purpose does it fulfil? We will admit it is here for the purpose of contraction, and that it does contract rythmically, that it does so for a purpose, and that the purpose is that the blood may be propelled into, or even through, the capillaries which have no such coat, and, therefore, no such power. I will now mention one experiment, which seems to show these vessels are really under the control of the organic nervous system:—

If the sympathetic nerve be divided on one side, in the neck of a rabbit, immediately afterwards the small arteries in the conjunctiva and in the ear are seen to become dilated, congestion, in fact, results, just as we would suppose it would, by removing from the vessels the regular or rythmical supply of motor stimulus, by dividing the nerve leading to them, by which the exciting power transmitted, on which the contraction of the vessels depends. We will then admit the small arteries contract rythmically under an influence afforded by the organic nervous system. If this is true, it follows if this nerve stimulus, for any reason, should be supplied in great excess, in the same proportion would these small arteries contract beyond what is normal; or if, on the contrary, we suppose this stimulus for any reason is withdrawn, either partially or wholly, from the vessels of any part, the effect would be their paralysis with consequent relaxation and distension or *congestion*. In so far, then, as the assistance of these vessels is necessary in the circulation of the blood, or so far as they have been diminished in activity, just so far will the circulation be retarded. *This is*

*congestion*, the indispensable preliminary step to the *post mortem* changes which follow in due order, as I will undertake to show in another paragraph. This may all, in the analysis, be traced to some cause which shall so act on the organic nervous system, or some part of it, as to destroy partially, or entirely, its influence over the vessels of some part or parts in the way already referred to. The agent, whatsoever it may be, by which this state is brought about, is what I suppose to be the *essential cause* of the disease. That there *are* known agencies which act not in the same, but in a *similar* way, I deem it unnecessary to show; and that this cause should act particularly on that part of the nervous system which controls the cerebro-spinal circulation, seems to me not any more strange than the peculiar action of many medicines which act on special parts of the body. If we reject the one because we cannot afford a rational explanation, for the same reason we must reject the other, but which, as a fact, no one thinks of disputing. We will then suppose the essential cause of the disease so to act on the organic nervous system, or that part, more *particularly*, which controls the circulation of the cerebro-spinal axis, as in various degrees to destroy its influence over the same, namely, the small arteries which ramify *so richly* in the *pia mater* and ventricular walls of the brain, and which do not have, as the larger arteries do, an *elastic* but a *muscular coat.* I should expect, *a priori*, any consequence which would flow from this state of the vessels to be most plainly marked where the vessels are most abundant, or on those parts of the pia mater most richly supplied with vessels, namely, parts at the base of the brain, along its fissures, sides, and upper surface of the brain, and on the medulla oblongata, and cervical and lumbar portions of the cord. This accords *entirely* with the *facts* of pathological anatomy.

Now, I suppose this poison to be capable, sometimes, of acting so energetically in destroying the excitant energy of the organic nervous system, and probably directly on the brain and spinal cord, as to produce death so soon that no considerable traces of disease shall be left to be observed after death, sufficient to account for the fatal result. Again, I suppose it may so act as to produce disorder of the capillary circulation, especially that of the cerebro-spinal axis, in the way already indicated. In various degrees its controlling influence over the circulation is destroyed. There is paralysis, more or less complete, of the muscular coat of the small arteries, the chief function of which, aside from that of a mere conduit, I believe to be to communicate a new impulse to the blood, *necessary* to circulate it through the capillaries. What I suppose to take place in the pia mater, *e.g.*, is *similar* to what *is observed* to take place in the conjunctiva or ear of a rabbit when the influence of the organic nervous system is withdrawn from the muscular vessels. I suppose the same phenomena to occur here as in the mesentery of a frog or the wing of a bat, at or toward the close of the experiments detailed. The vessels cease to contract, they relax, increase in size, not only for this reason permitting the blood to accumulate, but because they have lost the power to propel the blood onward, which is necessary to circulate it through the capillaries, which latter are entirely passive, as regards the circulation of the blood.

This is what I understand as *congestion*, and it results *not* from more blood being *sent* to the part, but because of increased size or capacity of the vessels, and because it is *detained* there through failure of the propulsive power of the small arteries. Blood is *constantly sent* to the part, but imperfectly sent away. Two conditions now favor transudation:—1st. *Stretching* or *thinning* of the *walls* of the relaxed vessels. 2d. *Increased expansive pressure from within outward, because* of a constant increase in the *volume* of the *blood.* I suppose the serous or watery part of the blood to exude *first.* Such is also the teaching of morbid anatomy. In it, and more especially a little later, the more solid elements of the blood, as the *fibrine* and *albumen*, or even the *corpuscles* at last. The blood thus detained in the vessels and made *viscid* by *loss of fluid*, I suppose to *coagulate*, and to be made here and there *utterly incapable* of being circulated, in this way permanently *obstructing* the vessels. The trouble once fairly initiated, it is easy to see how the effusion may be increased, how nutrition of the brain and cord will be interrupted, and how, through this and the *pressure* caused by the effusion, etc., we may have produced many of the symptoms of this disease or even death. In this same way, would I account for most effusions or exudations *anywhere*, either in the tissues or on free surfaces, whether in this disease or others. This distension of the small vessels I suppose may go on, determined by many circumstances, in some parts, until the vessels shall give way and true hemorrhages occur here and there, especially in the tissues, and thus the ecchymosed spots seem many times produced.

The changes which the effused matter will undergo, will depend, of course, on a variety of circumstances, which it is not our purpose to enquire into here. I do not deem it necessary to suppose the muscular coat of the small arteries, in *this* case, to have lost the *power* to contract, but simply that the excitant they normally should receive from the organic nervous system to have failed.

It seems to be the province of the cause of this disease to produce a state of depression or sedation of the nervous system, especially the organic. While there are many known agencies capable of producing sedative effects on this nervous system, the peculiar character of this cause seems to be that of acting in a marked manner on that part of the sympathetic system which controls the circulation of the cerebro-spinal axis.

At this time and in this paper, it would be manifestly improper to go farther in adducing proof that this explanation is a reasonable, perhaps a true one. I should not have entered on the preceding statements, if they had not seemed absolutely necessary to enable me to explain what I have learned to regard as the pathology of the disease. I would gladly have referred to some work in which the views glanced at in relation to the circulation are, especially in the small vessels and the relations sustained thereto by the organic nervous system, given, but I have not seen a satisfactory statement of such views in any author. I have examined these questions with some care, as shown elsewhere. They are introduced incidentally only. In a paper just referred to, I have examined, briefly, the principles glanced at in this discussion, and have shown their applicability in the explanation of many pathological states, as well as in the present case.

PATHOLOGICAL DEDUCTIONS, ETC.

In that part of the discussion over which we have passed, we seem to have reached the following conclusions:—

1. The symptoms which refer to the nervous system, while they vary, are, considered as a group, never absent. Other symptoms are mainly incidental.

2. The pathological appearances found in the cerebro-spinal cavity are, in some degree, always present, generally in a marked degree. Other pathological appearances mainly incidental. Contrary to the general opinion, it seldom attacks the serous surfaces within the cerebro-spinal cavity or elsewhere.

3. The blood before death uniformly shows a bright crimson color, coagulates rapidly and firmly, and often exhibits a decided buffy coat. Upon examination, it shows a decided increase of fibrine and, generally, of corpuscles. After death, the blood is generally, but by no means always, darker and more fluid than customary.

4. It is *not* a form of typhus.

5. It is probably neither contagious nor infectious.

6. It is probably due to a special external epidemic cause.

7. This supposed cause probably acts on the nervous system directly, perhaps primarily, whatsoever its action on the blood, so powerfully sometimes as to destroy life immediately, at others so as to induce, through the organic nervous system, disorder in the *circulation* and organic processes *dependent* on this nervous system, of the former, in a constant, special, and marked manner in the brain and spinal cord, entailing a series of consequences, where time is given, in this way, and probably by direct action of the cause on the blood, sufficient at last to produce death, independent of the real cause of the disease.

That the nervous system may recover its energy after the first impression of the poison, but through certain consequences, as effusion into the cerebro-spinal cavity, the disease may be continued for weeks or even months, either to death or a slow and painful recovery.

8. That the blood is probably not primarily diseased but becomes notably changed in some respects *during* the *progress* of the disease, and, doubtless, contributes much after a time to the unfavorable or fatal results in cases which have some duration. That the state of the blood is, in some measure, possibly due to the direct action of the poison, but seems to be mainly due to the disorder of circulation and nutrition, induced by the above mentioned state and influence of the organic nervous system.

9. The disease may take a distinctive hue or character, so to speak, from the prevailing forms of disease, as erysipelas, malarial or periodical diseases, typhus, etc., and that it may be complicated with various *special* forms of disease.

10. That it *is* a *cerebro-spinal meningitis*, if we name the disease from the standpoint of *a group* of its *most obvious* and *constant phenomena* or *effects*, and all other titles given from similar standpoints, as "spotted fever," deserve to be abandoned for obvious reasons. That it is impossible, at this time, to give the disease a name from the standpoint of *causation* which shall be satisfactory.

### THERAPEUTICAL DEDUCTIONS.

We seem to have two classes of cases in which the indications for treatment are somewhat different. Other subdivisions, more or less useful, have been or might be made, but the two classes here given seem to comprehend most, if not all, cases. They are:—

(*a*) 1. Those in which the cause operates with such intensity as to destroy life directly, before time has been given for pathological changes of any considerable importance to occur.

2. Those in which death does not occur directly from the effects of the poison, but in which the nervous system recovers from the first impression, and in which, time being given, pathological changes occur, as within the cerebro-spinal cavity or in th blood, many times sufficiently marked to become the cause of death or a protracted recovery, independent of the agency of the primary cause, and which cases at last show much of the character of ordinary inflammation of the contents of the cerebro-spinal cavity, are often sthenic or febrile in character, and require a quite different therapeutical management, in many respects, from those of the first class.

(*b*) If the views we have adopted be correct, then, upon well-recognized principles in therapeutics, we should be led, *a priori*, to a course of treatment in general as follows. In view of the first class of cases, and the actual phenomena of the disease, as they appear at an early period, we would turn:—

1st. To such known agencies as would efficiently stimulate the organic nervous system, such as opium, cantharides, camphor, chloroform, strychnia, the active principles of coffee and tea, quinine, brandy, etc.

2d. To such external agencies as would prove revulsive, by promoting the circulation at the surface of the body, and which would at the same time prove indirectly stimulant to the nervous system, such as blisters, heat, frictions, stimulating embrocations, and sometimes the alternation of heat and cold, dry cups, local bleeding occasionally, faradisation of the skin, etc.

3d. We would not, *a priori*, approve of general blood-letting or any general sedative measures at this stage, only in exceptional cases, nor would we in this stage depend much on alteratives, such, *e.g.*, as the mercurials.

4th. In the second class of cases, we would expect internal stimulants not to answer so well as in the first class of cases, and in the early stages of the disease. External stimulation, as blistering, we would expect would prove useful over the back of the head, neck, and spine. We should be led to think more of the application of cold, of blood-letting, both general and local, and of alteratives, such as the mercurials, iodide potassium, bromide potassium, etc., than in the former cases.

Such, in brief, are some of the therapeutical deductions, from the views we have arrived at, concerning the nature of the disease.

THERAPEUTICAL HISTORY.

It now remains for us to consider the *therapeutical his-*

*tory* of the disease, and in so doing, not only to determine what the treatment actually *has* been, but what has been the most *successful* treatment, and how far it agrees with that which, in the light of general principles, we have deduced from pathological considerations, and still farther try and see what light from its therapeutical, can be thrown back on its pathological history. Keeping in view the two great classes of cases already referred to, I will consider the principal remedial agencies which have been employed in this disease.

I. SEDATIVES.—In a general way, they have not been found useful in the early part, but have found more favor in the latter part of the disease, and in cases distinctly febrile.

1. *Blood-letting.*—Mons. MATTOT, of Auch, BASSERON, of Alger, CORBIN, of Orleans, TOURDES, of Strasbourg, MAILLOT, of Lisle, and others, have bled very freely from the arm, and by cups and leeches, but lost more than 60 per cent of their cases. They generally bled early, and the bleedings were often repeated in some cases, but with unsatisfactory results. Dr. DRAPER, however, and those to whom he refers, seem to have derived advantage from local bleeding. Drs. MINER and TULLY, and Dr. KEMPF, are decidedly opposed to blood-letting on practical grounds. The same conclusion has been reached by HIRSCH and RUMMEL, in the Dantzig epidemic. They repudiate general, but in some cases have resorted to local bleeding, apparently with good effects. Much the same is the testimony of NIEMEYER. BROUSSAIS would, however, bleed in the beginning from the arm, in most cases, and resort subsequently to local bleeding in the most active cases. FORGET, LEVY, and some others, would bleed locally, and in some of the most sthenic cases would resort to general bleeding. And so, in general, has it been with the majority of observers whose experience has been sufficiently broad to be useful in guiding us to correct conclusions. The weight of experience is against its employment, except with great caution, and principally in the latter part of the disease. Local bleeding, by cups and leeches, has most favor.

2. *Cold.*—Cold is here considered only in its primary effect.

While some have found cold to the head and spine not useful, the majority of observers, perhaps, have had apparently satisfactory results. Its application is attended with most benefit after reaction, and in cases decidedly febrile in character, while early in the disease, and in those cases marked by extreme depression, it has not seemed to be attended by happy results. Among those who have found it useful, may be mentioned RUMMEL, who has found it good, almost from the beginning, in cases where the face was flushed, the head hot, and where there was much pain; but in cases where the face was pale, not much pain, stupor, and the pulse low, he has not seen it followed by good effects, but the contrary. HIRSH and NIEMEYER report much the same. PANTHER liked the effect of a cold shower after a hot bath. FOVILLE, BROUSSAIS, ROLLET, and others, both in this country and Europe, concur, in the main, in the statements just made. The employment of cold, seems to have been most favorable early in the disease, when opium has been administered internally.

3. What has been said of blood-letting may be said of other sedative agencies, such as veratrum, aconite, belladonna, stramonium, and other remedies which have a similar action with them. But few or none of them have had an extensive trial, but so far as they have been tried, they have not yielded encouraging results. Belladonna has, perhaps, the most evidence in its favor. In active or sthenic cases, such remedies have generally proved useful, if I rightly collect the scattered testimony concerning their use. Digitalis, if it can in all cases be considered sedative, has seemed to give some good results. RUMMEL speaks highly of its use in the first few days of the disease. ROLLET speaks favorably of laurel water, and MIAHLE of hydrocyanic acid. Such, in general, seem to be the results of experience in the use of sedative agencies in this disease.

II. STIMULANTS.—They have been found useful at all periods of the disease, especially in proportion as they are early employed, both external and internal.

(*a*) *Internal.* 1. *Opium.*—This stands not only at the head of the list of stimulants, but also at the head of remedial agencies in this disease. There is none other, concerning which there is such unanimous and emphatic testimony. It has been employed in all stages, and in all kinds of cases, but has, generally speaking, proved most efficient in the early part of the disease, and in those cases which display the least acuteness in character. RUMMEL found it most efficient where the face was pale, pupils dilated, pulse low, etc., and in acute cases, if cold was freely applied to the head at the same time. But where copious exudation had occurred, it seemed to be of no advantage. It was very effective in allaying the sympathetic vomiting so often observed early in the disease. M. CHAUFFARD, of Avignon, found it highly beneficial. Dr. W. H. DRAPER gives very decided testimony as to its value, and assuming the disease to be a form of arachnitis, thinks the opium proves good here, as it does in peritonitis, since they are both inflammations of serous surfaces. I have, however, shown conclusively, that it is *not* an inflammation of the arachnoid. The most satisfactory results have been reported by MINER and TULLY, LIDDELL, BOUDIN, HIRSH, FORGET, LEVY, and many others. BROUSSAIS employed it freely after bleeding. These observers, and with them others, deem it the *best* remedy we have, while some, as HIRSH, and, more particularly, MINER and TULLY, consider it almost a specific. NIEMEYER, however, did not obtain such favorable results, though subcutaneous injections of morphia proved beneficial in his experience. The testimony of many others in this country might be added, as well as from Europe, but in the majority of cases they only serve to confirm the good opinion already expressed. The indications for its use, when they are mentioned, or as they appear, agree with those laid down by RUMMEL, from his experience. Here, as elsewhere, in the therapeutical history, the details are left to the judgment and good sense of such persons as this report may chance to reach.

2. *Quinine.*—A few observers have found this remedy beneficial, and especially in paludal districts in this country and Europe. WALES and GERHARD, in a limited experience, found it beneficial. Drs. OTTMAN and BURR, of Carbondale, Pa., found it useful in a few cases. But the majority are against its employment. RUMMEL, who seems to have been a very judicious observer, never found it useful, only in the later stages of the disease, when the mind had become clear, and then only as a tonic. Such has been my own experience. Mons. LEONARD and DURAND favor its employment, but evidently in cases where malarial influence has obtained. But HIRSH, whose experience was very extended, and who is a very competent observer, believes it does no good at all. Much the same has been the experience of Mons. GASTE, CHAUFFARD, PASCAL, FORGET, TOURDES, SCHILLIZI, etc. On the other hand, M. GASSAUD lost only 2 cases out of 126, according to his report. This excellent result he attributes to the early and free use of quinia. On the whole, it seems to be useful as a stimulant only in those cases complicated with malarial disease, and at an early period, or as a tonic toward the close of the malady, after the mind is clear, and febrile symptoms have disappeared.

3. *Cantharides.*—This remedy, from its known stimulating effect on the organic nervous system, would seem to promise some benefit. It has not been often tried, but, so far as it has been, the results are favorable. The individual who seems to have had the most extended experience with it, is Prof. ALLEN, of Rush Medical College, of this city. In his experience, and that of some others, it has yielded good results, mostly confined to the early stages, and in cases exhibiting marked depression.

4. *Strychnia.*—Dr. H. NOBLE, in a report on practical medicine, made to the Illinois State Medical Society, details a highly favorable experience with this remedy. Others, as WALES and PALMER, found it quite beneficial. It would seem, its use was attended with the most advantage in the early part of the disease.

5. Camphor, Carbonate Ammonia, Musk, Brandy, Valerian, and the various antispasmodics, have been employed. HALL seemed to obtain benefit from camphor and brandy; GERHARD from brandy; and such has been the experience of several physicians practising in the West in our late epidemics. FORGET

did not observe any advantage to result from the use of camphor, musk, ether, etc.

(*b*) *External Stimulants.*—External stimulation has proved one of the most efficient means in the treatment of this disease. It has been found useful at all periods of the disease. At the head of the list of external stimulants, stands blistering and counter-irritation along the spine. A few observers have not seemed to derive advantage from their use, but the majority have. DAVIS, of Chicago, NOBLE, THACHER, WALES, ATLEE, of Penn., HIRSH, BROUSSAIS, FORGET, and others, have recorded the most favorable results, as to the employment of blisters from cantharides, at all periods of the disease. RUMMEL highly recommends their use, especially in the middle and later stages of the malady. M. ROLLET obtained excellent results from the actual cautery, freely applied along the spine, especially in low cases. The application of stimulating embrocations and of severe frictions, especially over the spine and on the extremities, and the application of heat, either moist or dry, especially to the extremities, early in the disease, seems to have been attended with marked advantage.

III. ALTERATIVES.—They have, generally speaking, not proved useful in the early part of the disease, but have been satisfactorily employed in the latter stages of cases which have some duration, and especially where exudation has occurred abundantly.

1. *Mercurials.*—While some have seemed to derive advantage from them, those who have had the most extended experience have not had favorable results. RUMMEL, HIRSH, FORGET, CHAUFFARD, BROUSSAIS, DRAPER, etc., decidedly oppose the use of mercurials. M. ROLLET would use them prudently. Much the same is stated by M. LEVY. NIEMEYER seems more favorably disposed to their use. They seem to have answered best in complicated cases, and especially those where the secretions are inactive, and in the latter stages of acute cases with exudation.

2. *Iodide and Bromide Potassium.*—The former has proved highly efficient in the latter part of cases where exudation has occurred. This was especially true in the case of HIRSH and RUMMEL. The bromide of potassium has been occasionally employed with apparent benefit. From its known power of controlling irregular action of the nervous system and its general tranquilizing effect, it would seem to justify a more full trial than it has had. It was tried with benefit in a well-marked case, by Dr. ROGERS, of this city. The permanganate of potash has seemed to be useful, but does not promise much, on account of its unstable chemical constitution.

IV. EMETICS.—I have seen but one author who recommends emetics. There does not seem to be but few, if any, cases in which they are indicated. In the beginning of some cases, it might be desirable to evacuate the stomach, but they are rare.

V. PURGATIVES.—They are often indicated, and have often been employed, sometimes with advantage, at others with no perceptible good, and in some cases apparently with harm. Mild purgatives or laxatives, to unload the alimentary canal, and afterwards to maintain the bowels in a soluble state, seem admissible, if not actually demanded. This, with a judicious use of tonics and a careful diet in the latter stages and during convalescence, constitutes the management found most successful, as I have been able to glean it from numorous observers, times, and places. I have aimed at general results, only in the therapeutical history, not deeming it within the province, as it was not possible, in the limits of this report, to give details of treatment nor specific direction.

By comparing the results of experience in the treatment with the prior therapeutical deductions, it will be found they agree as far as could be expected. This agreement goes to confirm the correctness of the views which led to the deductions just mentioned. I have only space *now* to refer to this agreement, and to say, I have endeavored to approach the examination of this interesting disease without a single prejudgment, and I have also endeavored to conduct my examination at least in full view of the *inductive method.*

I feel that if I had some more time, I might far better elaborate some things in this report, but the limited time in which it has to be rendered, and the difficulty and irregularity of receiving reports from Europe, has made it impossible to do much more.

With these remarks, the report is respectfully submitted.

